# Active robotic training improves locomotor function in a stroke survivor

**DOI:** 10.1186/1743-0003-9-57

**Published:** 2012-08-20

**Authors:** Chandramouli Krishnan, Rajiv Ranganathan, Shailesh S Kantak, Yasin Y Dhaher, William Z Rymer

**Affiliations:** 1 Sensory Motor Performance Program, Rehabilitation Institute of Chicago, Suite 1407, 345 E Superior Street, Chicago, 60611, IL, USA; 2 Department of Physical Medicine and Rehabilitation, Northwestern University Feinberg School of Medicine, Chicago, IL, USA; 3 Department of Physical Medicine and Rehabilitation, University of Michigan Medical School, Ann Arbor, MI, USA

**Keywords:** Gait training, Gait velocity, Hemiparesis, Visual feedback, Muscle Synergies, Muscle modes, Skill acquisition, Variability, TMS, PCA

## Abstract

**Background:**

Clinical outcomes after robotic training are often not superior to conventional therapy. One key factor responsible for this is the use of control strategies that provide substantial guidance. This strategy not only leads to a reduction in volitional physical effort, but also interferes with motor relearning.

**Methods:**

We tested the feasibility of a novel training approach (active robotic training) using a powered gait orthosis (Lokomat) in mitigating post-stroke gait impairments of a 52-year-old male stroke survivor. This gait training paradigm combined patient-cooperative robot-aided walking with a target-tracking task. The training lasted for 4-weeks (12 visits, 3 × per week). The subject’s neuromotor performance and recovery were evaluated using biomechanical, neuromuscular and clinical measures recorded at various time-points (pre-training, post-training, and 6-weeks after training).

**Results:**

Active robotic training resulted in considerable increase in target-tracking accuracy and reduction in the kinematic variability of ankle trajectory during robot-aided treadmill walking. These improvements also transferred to overground walking as characterized by larger propulsive forces and more symmetric ground reaction forces (GRFs). Training also resulted in improvements in muscle coordination, which resembled patterns observed in healthy controls. These changes were accompanied by a reduction in motor cortical excitability (MCE) of the vastus medialis, medial hamstrings, and gluteus medius muscles during treadmill walking. Importantly, active robotic training resulted in substantial improvements in several standard clinical and functional parameters. These improvements persisted during the follow-up evaluation at 6 weeks.

**Conclusions:**

The results indicate that active robotic training appears to be a promising way of facilitating gait and physical function in moderately impaired stroke survivors.

## Background

There has been a growing interest in using robotic therapy to improve walking ability in individuals following hemispheric stroke [1,2]. The primary reasons for utilizing robotic interventions are that appropriately designed robots are able to minimize therapist burden while providing large volume of task-specific practice in novel dynamic environments, and that they allow continuous monitoring of patient performance and progression [2,3]. These factors could enhance therapists’ productivity by reducing their workload and providing relevant information that is critical for clinical decision-making. In this regard, several rehabilitation robotic devices (for e.g., Lokomat, Electromechanical Gait Trainer, LOPES, ALEX, and Rutgers Ankle Rehabilitation System) have been introduced in the past decade to facilitate recovery of walking ability after neurological insult such as stroke or spinal cord injury.

Several studies have evaluated the efficacy of robot-aided gait therapy in improving gait and physical function after stroke [4-7]. While many of the robotic devices were successful in achieving the goals mentioned above [8,9], the rehabilitation outcomes were not significantly different and sometimes were inferior to manual therapist-assisted treadmill training or conventional rehabilitation approaches, especially in ambulatory stroke subjects [5,6]. This has generally been attributed to the substantial guidance provided by the robot (i.e., the robot assists the movement regardless of whether or not the subject intends to move) that not only leads to a reduction in volitional physical effort, but also potentially interferes with motor relearning [10-12].

In this case study, we tested the feasibility of a novel robotic gait training paradigm on a moderately impaired stroke survivor. The paradigm consisted of two components: (1) a patient-cooperative robot that allows participants to actively control the motion of the limb while providing minimal assistance, (2) a target-tracking task that involved matching the ankle position to a particular movement template. The findings from this case study suggest that active robotic training may be a promising means of mitigating gait impairments in stroke survivors.

## Materials and methods

### Subject

The subject was a 52-year old male stroke survivor (7 months post-stroke) who suffered right hemiparesis due to a left corona radiata infarct. Immediately after his stroke, he was admitted to an acute care hospital for five days, which was followed by 3-weeks of inpatient physical and occupational rehabilitation. He then received outpatient rehabilitation three times a week for the next four months. Before the initiation of the intervention program, the patient was able to ambulate in the community with the assistance of a cane and an ankle-foot orthosis. The subject revealed no major sensory or cognitive deficits (Mini-Mental State Examination Score of 29 [13]). He had normal range of motion in all the joints of the lower-extremity and showed no significant spasticity (Modified Ashworth Score ≤ 1). The subject provided written informed consent by signing a form that was approved by the Northwestern University Human Subjects Research Institutional Review Board.

### Intervention

The subject underwent active robotic training for 12 sessions (3 sessions per week for 4 weeks). During this period the subject did not receive any other therapy or participate in any other research opportunities. An orientation session was provided before the start of the intervention to familiarize the subject with the training conditions and with robot-aided walking. Each training session lasted for about 90 minutes (excluding set-up time of ~ 10–15 minutes) with several periods of rest provided, as needed. During each training session, the subject walked with the aid of a patient-cooperative robot and practiced a gait pattern that necessitated greater hip and knee excursion during swing phase of the gait cycle. No body-weight support was provided during training.

Active robotic training consisted of two components: (1) walking in the patient-cooperative control mode in the Lokomat that reduced the amount of guidance from the robot and (2) performing a target-tracking task during walking that involved matching the ankle trajectory (i.e., position of lateral malleolus in the sagittal plane) to a target-template in order to facilitate active participation from the subject. These components are described in detail below.

#### Patient-cooperative control in Lokomat

The Lokomat is a robotic device that has been widely used for gait rehabilitation in several movement disorders [14-18]. The Lokomat system consists of a powered gait orthosis with integrated computer controlled linear actuators at each hip and knee joint, a body-weight support system, and a treadmill. Traditionally, the Lokomat is configured to run in passive position control mode [19]. In this configuration, the Lokomat moves the legs of the subject along a fixed reference trajectory of the knee and the hip in a repeated cyclical motion in the sagittal plane with pre-determined gait parameters. This passive position control mode does not require active participation of the patient as the robot imposes a predetermined gait pattern irrespective of whether the patient attempts to move his/her legs. Moreover, even if the patient attempts to produce movements, the stiffness of the robot is so high that the patient has little influence on the imposed trajectory.

Recently, a patient-cooperative controller has been developed for the Lokomat, in which the stiffness of the robot can be programmatically controlled (from complete assistance to no assistance) so that the robot allows active participation of the patient [20,21]. Additionally, an adjustable virtual tunnel around the reference trajectory in joint space has been incorporated into the system using a path control strategy [20] so that the patient is in control of the spatial and temporal characteristics of the gait pattern. The virtual tunnel allows free movement as long as the leg postures are within the tunnel, but applies corrective torques if the leg position is outside the tunnel. The concept of virtual tunnel incorporates the motor learning principle of bandwidth feedback [22-24] as the patient receives feedback in the form of corrective torques only when the movement goes outside a tolerance zone around the reference trajectory [25,26]. The width of the virtual tunnel (i.e., the tolerance zone) is adjustable such that a narrow tunnel does not allow the patient’s trajectory to deviate much from the reference trajectory whereas a wider tunnel allows greater deviation before the robot assists the movement. The tunnel is also shaped in such a manner that it allows more variation during late swing and early stance phase. In order to ensure that the subjects have minimal interactions with the robot inside the tunnel, a transparency-enhancing potential force field using generalized elasticities [21] was used to mask the weight and inertia of the robot. The details of the control algorithms that describe both the path control strategy and generalized elasticities can be found elsewhere [20,21].

#### Target-tracking paradigm

**Figure 1:**
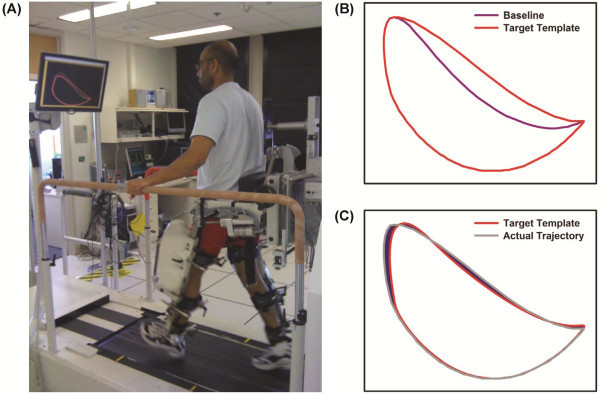
**(A) Schematic representation of active robotic training.** ( **B**) **Construction of target-template trajectory – the target-template trajectory was constructed from the ensemble average of the baseline hip and knee trajectory with a scaling factor of 0.2. The scaling factor represents the amount of additional hip and knee flexion angle required from the baseline gait (i.e., when there is no tracking involved). In this case, the subject had to increase his hip and knee joint angle by 20% to match this target-template. The actual trajectory represents the ensemble average of the baseline trajectory computed from the hip and knee joint angle recorded during the baseline walking(C)Computation of target-tracking error – the error during target-tracking was calculated by determining the area that was not common to both the target-template and the actual trajectory during each gait cycle (shaded region). The resulting area (i.e., error) was normalized to the area of the target-template in order to compute % error during target-tracking.**

Active participation was also facilitated by including a target-tracking task with the aid of visual feedback [27-29]. Once inside the Lokomat, the subject performed a target-tracking task while walking over a split-belt ADAL treadmill (Techmachine, Andrezieux Boutheon, France) with embedded force plates (Figure 1A). The subject was instructed to adjust his own ankle trajectory in order to match a desired target-template. It is important to note that the term ‘ankle trajectory’ refers to the position of the lateral malleolus in the sagittal plane while walking in the Lokomat and *not* to the movements of the subject’s ankle joint. The ankle position is uniquely determined by the hip and knee joint angles (see below for details) and therefore changes in this pattern will require changes in the hip and/or knee joint angle. Visual feedback of the subject’s ankle trajectory was provided during the training on a computer monitor placed in front of the subject [30]. The desired target- template was also displayed concurrently with the subject’s actual ankle trajectory and the subject was instructed to match the target by modifying the movement of his paretic leg.

The instantaneous ankle position (*x *_*a*_*, y*_*a*_) of the subject was computed from the hip and knee joint angles recorded with the potentiometers inbuilt in the Lokomat using the following equation:

(1) $$\left[\begin{array}{c} x_{a}\\ y_{a} \end{array}\right]=\left[\begin{array}{cc} sin\theta_{h} & -sin\left(\theta_{k}-\theta_{h}\right)\\ -cos\theta_{h} & -cos\left(\theta_{k}-\theta_{h}\right) \end{array}\right]\left[\begin{array}{c} l_{1}\\ l_{2} \end{array}\right]$$

where *l*_1_ is thigh segment length, *l*_2_ is shank segment length, *θ*_*h*_ is hip joint angle, and *θ*_*k*_ is knee joint angle. The visual feedback was displayed so that the entire series of ankle positions for one whole gait cycle was visible on the screen (instead of just a single point).

##### Setting the target-template

The target-template was changed depending on the subject’s familiarity with walking in the Lokomat. During the initial 5 visits of training, when the gait inside the Lokomat was highly variable, the desired ankle trajectory was based on patterns built into the Lokomat (these patterns were based on joint excursions exhibited by healthy subjects in the Lokomat when walking over obstacles with a height of 3 centimeters [19]).

From the 6^th^ visit onwards, we provided a target-template that was based on the subject’s own gait pattern. The target-template was constructed as follows: First, we computed the baseline trajectory by ensemble averaging the paretic leg ankle trajectory at the beginning of each training session when the subject walked in the Lokomat for two minutes. Second, the target-template was set so that it corresponded to a gait pattern which required increasing the hip and knee joint angle of the baseline gait by a specified scaling factor. Because the ankle trajectory is constrained by the ground during the stance phase of the gait, the increase in hip and knee angle was performed only during the swing phase of the gait. A Hanning window was used to prevent abrupt changes in ankle trajectory due to this increase in hip and knee angle during the initial and final part of the swing phase. The target-template was obtained from the baseline trajectory by using the following formula:

(2) $$\left[\begin{array}{c} x_{\mathrm{target}}\\ y_{\mathrm{target}} \end{array}\right]=\left[\begin{array}{c} x_{\mathrm{ba}}\\ y_{\mathrm{ba}} \end{array}\right]+s\left[\begin{array}{c} x_{\mathrm{hw}}\\ y_{\mathrm{hw}} \end{array}\right]$$

where *x*_*hw*_, *y*_*hw*_ represent the Hanning-windowed version of the baseline trajectories (*x*_*ba*_*, y*_*ba*_) and *s* was the scaling factor (Figure 1B). The scaling factor was gradually increased from 0.1 to 0.3 from the 6^th^ visit to the end of training. After practicing this task for about an hour, the subject also practiced matching a second target-template that was derived from the patterns of his less impaired leg (i.e., non-paretic leg) for about 20 minutes. This second template was used to practice gait patterns that were closer to normal gait as the information regarding the optimal gait pattern to train a stroke subject to achieve maximal recovery is currently not known.

We chose the greater hip and knee excursion pattern for most part of the training due to three reasons. First, we wanted to target the characteristic stiff-knee gait (which arises due to difficulty in producing adequate hip and knee flexion during swing phase of the gait) that is seen in many stroke survivors including the subject that participated in our study. Second, we believed that this pattern will not only increase active participation, but would also increase the physical effort required by the subject to walk in the Lokomat thereby preventing the “slacking phenomenon” and promoting strength and endurance. Finally, previous research that has incorporated a similar approach of training has shown positive outcomes in patients’ walking capacity in the treadmill [28].

##### Progression of task difficulty

The progression in training was achieved by increasing the subject’s walking speed in the Lokomat and by reducing the visual feedback during target-tracking (Table 1). The speed at which the participant trained was gradually increased within and between sessions, starting at 2 km/hr at the beginning of training (session 1) and reaching a maximum of 3.5 km/hr at the end of training (session 12). The initial walking speed and the criteria for its change were based on self-reported patient’s tolerance in the Lokomat. The subject received continuous real-time feedback of his ankle trajectory during the initial 4 visits. Thereafter, visual feedback was gradually reduced and was only provided when the subject was deviating considerably from the desired target-template. Deviations from the target-trajectory were visually identified by the experimenter since the numerical analysis could not be performed in real-time.

**Table 1:** Progression of training over the 12 treatment sessions

**Session**	**Speed**	**Scaling Factor**	**Visual Feedback**
1	2 Km/hr	0	Continuous
2	2.0 – 2.5 Km/hr	0	Continuous
3	2.0 – 2.7 Km/hr	0	Continuous
4	2.0 – 2.7 Km/hr	0	Continuous
5	2.0 – 3.0 Km/hr	0	Intermittent
6	2.2 – 3.0 Km/hr	0.1	Intermittent
7	2.2 – 3.2 Km/hr	0.1	Intermittent
8	2.2 – 3.3 Km/hr	0.2	Intermittent
9	2.4 – 3.5 Km/hr	0.2	Intermittent
10	2.4 – 3.5 Km/hr	0.25	Intermittent
11	2.7 – 3.5 Km/hr	0.25	Intermittent
12	2.7 – 3.5 Km/hr	0.3	Intermittent

Note the scaling factor was ‘0’ in the initial 5 visits as the patient was trained using the desired ankle trajectory based on kinematic patterns that has been built into the Lokomat, which already necessitates the subject to walk with greater hip and knee excursion.

### Outcome measures

Neuromotor performance and recovery were assessed at multiple levels – biomechanical, neuromuscular and clinical. All testing was performed without the use of cane and ankle-foot orthosis.

#### Biomechanical level

At the biomechanical level, we evaluated target-tracking performance and kinematic variability of ankle trajectory during robot-aided treadmill walking. We also assessed if the training transferred to overground walking by measuring the ground reaction forces (GRFs) generated during overground walking.

##### Target-tracking performance

The ability of the subject to accurately track a given target-template trajectory was evaluated by determining the area that was not common to both the reference trajectory (i.e., the target-template) and the actual trajectory during each gait cycle (Figure 1C). The area was computed using image processing techniques in MATLAB (Mathworks, Natick, MA) by converting the reference and actual trajectories into binary images and counting the number of pixels that were not common to both images. The resulting area (i.e., error) was normalized to the area of the reference trajectory in order to compute % error during target-tracking. The error values were then averaged across all the gait cycles collected during a 2-minute robot-aided walking to determine the mean error during target-tracking. The target-tracking performance was evaluated during the initial and final part of target-tracking practice at various time-points (early, post-intervention, and follow-up). The amount of assistance provided by the robot was kept constant (at the 10% guidance setting) for all of the testing sessions. Although the template was modified only during the swing phase of the gait, we computed the error during the entire gait cycle as changes in ankle trajectory during the swing phase might also involve changes in the trajectory during the stance phase.

##### Kinematic variability of ankle trajectory

In addition to the error, we also computed the kinematic variability of the ankle trajectory from the aforementioned target-tracking data (target-tracking variability) and from a 2-minute baseline walking data (baseline variability) collected during the pre, post, and follow-up testing sessions. The variability was quantified similar to the error computation described above, except that instead of the target-template, the ensemble average of the ankle trajectory during baseline walking (for baseline variability) or target-tracking (for target-tracking variability) was used as the reference. The resulting area from each gait cycle was normalized to the area of the reference trajectory to determine% deviation. The values from all the gait cycle were averaged to find the overall% deviation.

##### Ground reaction forces

GRFs were measured during the stance phase of the gait cycle for both the paretic and the non-paretic legs while the subject walked at his self-selected speed along a 10 m walkway equipped with instrumented force plates (AMTI, Watertown, MA). The GRF data were sampled at 1000 Hz and low-pass filtered at 10 Hz using an 8^th^ order Butterworth digital recursive filter. Four to six trials were collected for each leg and were ensemble averaged for further analysis.

#### Neuromuscular level

At the neuromuscular level, we evaluated muscle coordination using surface electromyography (EMG) and motor cortical excitability (MCE) during treadmill walking.

##### Muscle coordination

In order to assess muscle coordination, bipolar EMG electrodes were placed over the muscle bellies of vastus medialis (VM), rectus femoris (RF), medial hamstrings (MH), lateral hamstrings (LH), tibialis anterior (TA), medial gastrocnemius (MG), soleus (SO), and gluteus medius (GM). A common ground electrode was placed over the patella.

The EMG signals from each muscle were amplified (×1000), filtered (20-500 Hz) and stored for off-line analysis using an AMT-8 Bortec EMG system (Bortec Biomedical, Calgary, AB). These were then digitally high-pass filtered (40 Hz), rectified, and smoothed using a 2^nd^ order Butterworth filter (4 Hz).

The muscle coordination during walking before and after training was analyzed using dimensionality reduction techniques. The EMG from each stride was interpolated into 101 points and the resulting N x 8 matrix (where N = n × 101, n is the number of strides) was subject to a principal component analysis (PCA) followed by a varimax factor rotation. A minimum of 25 strides were collected during each session. The PCA analysis effectively expresses the muscle activity as the linear combination of a smaller set of muscle modes, each having time varying activations [31,32]. In essence, this captures the temporal coordination between muscles and a single muscle mode contains muscles that are activated at similar phases in the gait cycle (e.g., medial and lateral hamstrings). The amount of variance accounted for by these muscle modes captures the degree of coordination (i.e., if the muscle activity of the 8 muscles were completely independent of each other, then 4 muscle modes would capture only 50% of the variance). We extracted 4 muscle modes from the data as previous work suggests that 4 modes are sufficient to capture unilateral lower-extremity muscle activation data with a similar number of muscles [32]. We then compared these muscle modes to those obtained in young healthy individuals by computing the correlation coefficient for each muscle modes and taking their average (using a z-transform). The data from healthy controls were obtained from 6 males (age: 30 ± 4 years) walking on a treadmill as part of an earlier study [33].

##### Motor cortical excitability

In order to examine if training induced motor cortical plasticity, we assessed MCE by comparing the motor evoked potentials (MEPs) elicited with transcranial magnetic stimulation (TMS) before and after the intervention. MEPs were not assessed during the follow-up evaluation due to technical difficulties. MEPs were recorded from the subject’s paretic leg with the EMG electrodes on the 8 muscles mentioned above.

TMS was delivered using a Magstim 200 stimulator (110 mm double-cone coil) over the primary motor cortex (M1) of the left lower-extremity as the patient walked on a treadmill at 2 km/hr. The coil was centered 2 cm posterior and 1 cm to the left of the vertex and was securely attached to the subject’s head using Velcro [34,35]. A pulley system was used to reduce the weight of the coil and the cable on the patient’s head. TMS was applied during treadmill walking at precise instants of the gait cycle using a custom-built photosensor and a delay circuit that allowed for fine-tuning of the trigger-timing. For GM, VM, RF, MG, and SO muscles, MEPs were elicited during the stance phase of the gait cycle, while for the MH, LH and TA muscles, MEPs were elicited during the swing phase. TMS was applied during randomly selected gait cycles and timed to the onset of EMG burst of each muscle. The TMS intensity was tuned specific to each muscle such that the MEPs elicited were clear and distinguishable from the background EMG activity [34]. The same TMS intensity was used pre- and post- intervention and 12–15 MEPs were collected for each of the muscles. MEP data were analyzed using Spike2 software (Cambridge Electronic Design, Cambridge, England). MEP amplitudes were expressed as a percentage of the background EMG activity that immediately preceded the TMS pulse.

### Clinical measures

In addition to our biomechanical and neuromuscular measures, subjective and objective clinical outcome measures were assessed at 3 time points: (1) prior to training, (2) after completing 12 training sessions, and (3) 6-weeks after training (follow-up visit) using standardized procedures. These measures included the self-reported recovery in Stroke Impact Scale (SIS), Timed Up-and-Go (TUG) test, 6-minute walk test for distance, self-selected and fast walking speed, single leg balance test, and lower-extremity Fugl-Meyer score. Self-reported recovery in SIS was evaluated by having the patient report on a scale of 0 to 100 (with 100 representing full recovery and 0 representing no recovery) how much he has recovered from his stroke. Walking speed was measured while the subject walked on a Gait Mat II computerized measuring system (3.84 m x 0.6 m) at self selected and maximum possible walking speeds. Three trials were performed and the average of the three trials was used in the analysis.

## Results

We first describe the changes observed at the biomechanical and neuromuscular levels followed by the changes observed at the clinical level.

### Target-tracking performance & kinematic variability of ankle trajectory

**Figure 2:**
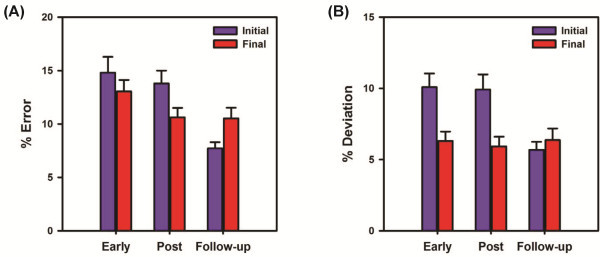
**Bar graphs representing the changes in (A) target-tracking error and (B) target-tracking variability with training.** The target-tracking error and variability were evaluated from the data (2-minute block) collected during the initial and final part of target-tracking practice at various training sessions (early – 2^nd^ session, post-intervention, and follow-up). Error bars represent 95% confidence intervals (between-stride). Note that the subject was able to reduce the target-tracking error and variability with practice, even though the templates for target-tracking were different at the 3-time points.

**Figure 3:**
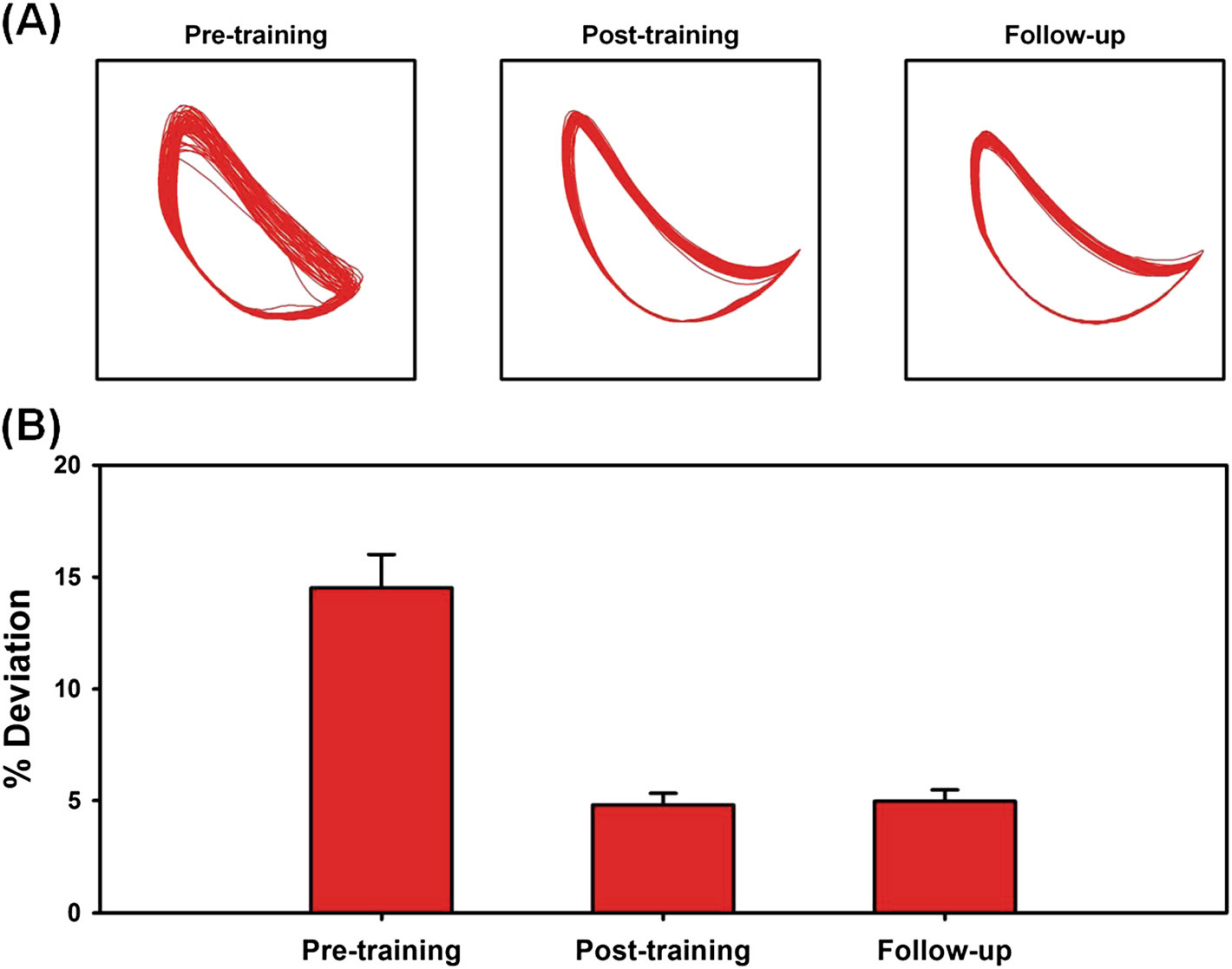
**(A) Ankle trajectory of the paretic leg during baseline walking at the three time-points of evaluation. (B)Bar graphs representing the kinematic variability of the ankle trajectory during baseline walking.** The variability was calculated by determining the area that was not common to both the ensemble average of the baseline ankle trajectory and the actual baseline trajectory during each gait cycle. The resulting area was normalized to the area of the ensemble average of the baseline trajectory in order to compute% deviation. Error bars represent the 95% confidence intervals (between-stride). Note that the variability of the ankle trajectory reduced by about two-folds with training and was maintained at the follow-up evaluation.

The target-tracking error decreased with practice during early- and post-training testing sessions (Figure 2A). With practice, the subject was able to reduce the target-tracking error by about 28% from early training (session 2) to the end of training (post-training), and this reduction was maintained during the follow-up evaluation (Figure 2A). The target-tracking variability followed a trend that was similar to target-tracking error. With practice, the target-tracking variability was reduced by 41% from the early-training to the end of training (Figure 2B), which was maintained during the follow-up evaluation. The baseline variability when walking in the Lokomat (when there was no tracking involved) also decreased considerably after 4 weeks of active robotic training (Figure 3A). There was a two-fold reduction in baseline variability after training, which was also maintained during the follow-up evaluation (Figure 3B).

#### Ground reaction forces

**Figure 4:**
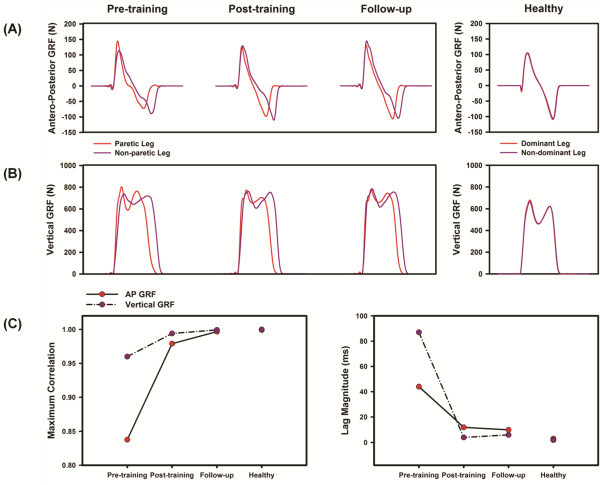
**(A) Antero-posterior (top panel) and (B) vertical ground reaction forces (middle panel) recorded during overground walking.(C)The bottom panel represents the changes in maximum correlation and lag magnitude (ms) obtained from the cross-correlation analyses between the paretic and non-paretic leg antero-posterior and vertical ground reaction forces.** For comparison, GRF data from healthy adults that have been published elsewhere are shown on the right column. Note that the two measures of between-leg symmetry showed a trend toward greater symmetry (increase in maximum correlation, decrease in lag magnitude) with training.

Figure 4A &4B illustrates the antero-posterior and vertical GRF traces of the subject during overground walking. For comparison, we have also provided data from 6 healthy adults that have been published elsewhere [36]. The subject exhibited substantial asymmetries in both the vertical and antero-posterior GRF patterns before training. Cross-correlation analyses indicated that the GRF patterns between leg became more symmetric (as noted by the increase in maximum correlation and decrease in lag magnitude) after training and continued to improve during the follow-up evaluation (Figure 4C). The maximum correlation and lag magnitude values observed after training were also closer to those observed in healthy adults (Figure 4C). Training also resulted in substantial increase in the peak propulsive force of both the paretic (35%) and the non-paretic leg (24%). These improvements persisted during the follow-up evaluation (paretic leg: 47%, non-paretic leg: 17%).

#### Muscle coordination

**Figure 5:**
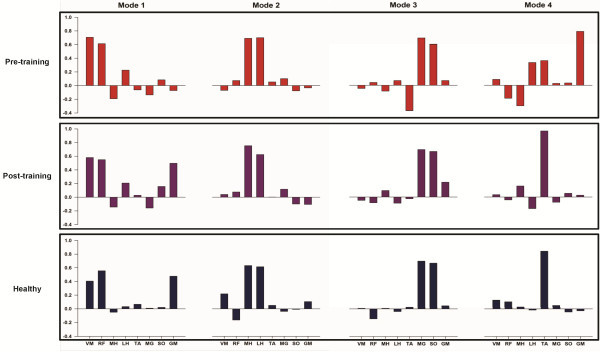
**Composition of muscle modes during treadmill walking.** The top and middle rows show the muscle modes for the subject pre- and post- training. For comparison, muscle modes from healthy controls are shown on the bottom row. Muscle modes were extracted from the EMG data using PCA followed by a varimax rotation. Note that the muscle modes post-training closely resemble those of healthy individuals.

There was also a change in the composition of the muscle modes after training, with the muscle modes after training resembling healthy controls (r = 0.93) more than before training (r = 0.71). The main changes after training were due to the first and fourth muscle modes (Figure 5). The variance accounted for by the 4 muscle modes was also higher after training (R^2^ = .91) compared to before training (R^2^ = .85), showing that the motor output was highly coordinated after training.

#### Motor cortical excitability

**Figure 6:**
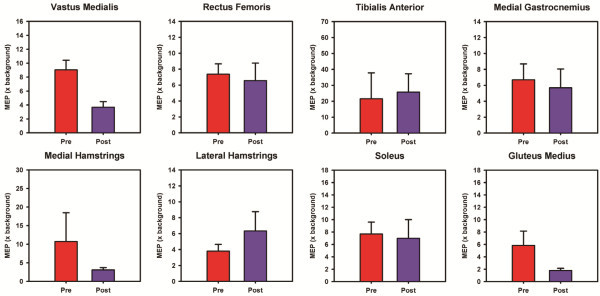
**Bar graphs representing the motor cortical excitability of the paretic leg muscles measured using TMS during treadmill walking.** The motor evoked potential (MEP) amplitudes were normalized to the background activity measured immediately preceding the TMS pulse. Error bars represent the 95% confidence intervals (between-stride). Note that the MEP amplitudes of vastus medialis, medial hamstrings, and gluteus medius muscles reduced substantially after training.

The mean TMS elicited MEPs of the lower-extremity muscles during treadmill walking are provided in Figure 6. The MEP amplitudes of the VM, MH and GM muscles decreased after training (Figure 6), whereas the MEP amplitudes of the other muscles remained about the same after training.

#### Clinical outcomes

There were large improvements in all of the measured subjective and objective clinical outcome measures (Table 2). The subject’s locomotor ability improved after training as reflected by positive changes in his walking speed, 6-minute walking distance and time taken to complete the TUG test. Most of these improvements were sustained during the follow-up evaluation. Training also resulted in substantial increases in single-leg balance, lower-extremity Fugl-Meyer score, and self-reported percent recovery in stroke impact scale.

**Table 2:** Clinical outcome measures before and after training

**Performance Variable**	**Pre-training**	**Post-training**	**Follow-up**	**1 SEM**
Timed Up-and-Go test (s)	14	11	11	1.14
6-min walk test (m)	228	316	304	18.6
Single-leg balance (s)	1	15	14	NE
Self Selected walking speed (m/s)	0.72	1.0	.85	.07
Fast walking speed (m/s)	1.1	1.3	1.3	.08
Stroke Impact Scale - Recovery	50	70	65	NE
Lower-extremity Fugl-Meyer Score	16	20	23	3.2

SEM = standard error of measurement established in the literature for the stroke population, NE = not established for stroke population Note that the changes observed after training are fairly larger than the 1 SEM values (which is a common coefficient used to assess whether the changes are clinically meaningful) established in the literature for stroke population.

## Discussion

In this case study, we tested whether facilitating active participation of the patient while receiving robot-aided gait therapy would help to mitigate gait impairments after hemispheric stroke.

Our preliminary evaluation indicated that four weeks of active robotic training led to substantial improvements in several clinical and functional parameters and these improvements persisted during the follow-up evaluation. Importantly, the improvements were much larger than the measurement errors [37] and were also substantially larger than the mean improvements seen after conventional Lokomat assisted gait therapy [6,17,38] and manual therapist-assisted treadmill training [6,17,39]. The positive clinical outcomes observed also reinforce the findings of a recent study that evaluated kinematic changes after robot-aided gait therapy using a similar approach [28]. Although we are limited by the fact that we have studied only a single subject, the results suggest that these outcomes were clinically meaningful (see Table 2) and that patient cooperative robot-aided gait training with target-tracking is a feasible and *potentially* effective approach to minimizing post-stroke gait impairments.

In addition to improvements that were specific to training (like reduced tracking error and variability), our results also showed improvements in abilities that were not specifically trained. First, our subject showed an improvement in single-leg balance on the paretic leg, going from 1 s before training to 15 s after training. This improvement was also sustained during the follow-up exam. The increase in single-leg balance time may be important from the viewpoint of reducing the risk of falls [40,41]. An interesting feature is that this improvement occurred despite the fact that the Lokomat restricts lateral and axial motion of the pelvis – an important feature for training balance and stability [42]. Second, there was transfer to overground walking as reflected by improvements in walking speed, the TUG test and the 6-minute walking distance. These improvements suggest that robotic training can produce improvements that are not simply context-specific, but are generalizable to activities of daily living.

We also used several novel biomechanical and neuromuscular metrics to complement the typical clinical measures, which allowed us to objectively quantify the mechanisms underlying the neuromotor deficits and monitor changes with training. At the biomechanical level, we found that training not only improved the propulsive forces generated by both the paretic and non-paretic leg muscles, but also improved the symmetry of the antero-posterior GRFs between the paretic and non-paretic legs (Figure 4). This finding suggests that positive outcomes observed after training were partly mediated by the improvements in motor output of both the paretic and non-paretic leg muscles and not simply due to functional compensation [43,44]. We also note that the changes in peak antero-posterior GRF of the paretic leg were much larger than the minimal clinically important changes that have been reported for this variable [45].

At the neuromuscular level, we found changes both in muscle coordination and motor cortical excitability during treadmill walking. In terms of muscle coordination, we found that the muscle modes obtained after training were closer to healthy controls, suggesting that part of the gait improvements were due to improvements in the timing of muscle activity. This was particularly evident in the timing of activation of the GM and TA muscles. In addition, the motor cortical excitability of the VM, MH, and GM muscles reduced substantially after training. The reduction in motor cortical excitability is probably due to improvements in lower-extremity muscle strength after training as previous studies indicate that strength training decreases motor cortical excitability [46-48]. While we do not have muscle-specific strength measurements to support this hypothesis, our subject reported improvements in strength and muscle control with training. Moreover, changes in propulsive forces and Fugl-Meyer scores suggest that our subject may have realized meaningful strength gains after training.

What are the critical components of the current training paradigm that may have facilitated the positive outcomes observed? As described earlier, the prevalent hypothesis for the suboptimal outcomes in several robotic interventions is that the guidance provided by the robot results in a lack of active participation during training [11]. Indeed, studies on motor learning show that excessive guidance may impair learning, even though it may augment performance temporarily [49,50]. Here, we incorporated a control algorithm that reduced the amount of passive guidance/support provided by the Lokomat. This ensured that the subject could not slack and instead had to actively walk inside the Lokomat. However, in addition to reducing robot guidance, we also added a skill-learning component by using a target-tracking task that forced the participant to exaggerate hip and knee flexion during the swing phase. We anticipated that this tracking task would not only result in increases in muscle activity, but would also challenge the subject to reorganize the muscular coordination in order to produce the new gait pattern consistently. Further, we also ensured that the visual feedback during this tracking task was perceptually simple by using the position of the ankle (in x-y coordinates), thereby making it easy for participants to identify and correct their errors (e.g., “I am not lifting my foot high enough”). This perceptual simplicity has been shown to be important for coordinating multiple degrees of freedom [51,52]. This feedback was also faded with practice in order to prevent the subject from being dependent on the visual feedback [53,54]. This type of task-oriented gait therapy, which involves both active participation and skill learning, may be critical in promoting better functional recovery in stroke individuals.

### Limitations

We would like to point out some of the limitations of this study. First, the results reported are from a single subject and it is possible that our subject may not be representative of the typical stroke population at large. He had only moderate levels of lower-extremity impairment, good upper-extremity function, and was highly motivated. As a result, he was able to cope well with the training regimen and perform the target-tracking task without much difficulty. However, stroke subjects with higher levels of impairment may not have adequate knee/hip flexor strength to perform this task. Although the guidance force exerted by the robot can be tuned specifically to match the recovery state and functional capacity of each subject, it remains to be seen whether these improvements will be observed in patients with higher impairment levels. Second, some of the benefits observed in our subject could have been due to natural biological recovery, although this seems unlikely given the evidence that most of neurological and functional recovery plateaus after 3–4 months following stroke [55]. Furthermore, it is possible that part of these improvements observed could have been solely due to the benefits of treadmill walking and not due to the robot-aided gait therapy. However, it is to be noted that the changes in functional outcomes observed in our subject is greater than those observed in any of the stroke subjects who participated in a recent pilot study that evaluated the effectiveness of manual therapist assisted treadmill training in stroke survivors [39]. Finally, there is also the question of whether it is the visual feedback or the cooperative control that was responsible for the improvements. In this regard, a study from Kim et al. [30] suggests that the combination of visual feedback and cooperative control is better for motor learning than either one alone.

### Summary

In this case study, we report results from a novel robotic gait training approach that aimed to facilitate active participation and mitigate post-stroke gait impairments. Four weeks of patient-cooperative robot-aided walking with a target-tracking task resulted in clinically meaningful improvements in several of the measured locomotor outcomes. These improvements persisted during a follow-up evaluation that was performed at 6-weeks after the completion of training. The promising positive outcomes of this case study suggest that combining patient-cooperative robot-aided walking with a target-tracking task is a feasible approach to improve post-stroke walking function. Further research is necessary to identify whether this approach would be feasible in patients with varying levels of impairment and also to verify whether similar results can be obtained from a larger cohort of stroke population.

## Abbreviations

GRF, Ground reaction force; EMG, Electromyography; MCE, Motor cortical excitability; VM, Vastus medialis; RF, Rectus femoris; MH, Medial hamstrings; LH, Lateral hamstrings; TA, Tibialis anterior; MG, Medial gastrocnemius; SO, Soleus; GM, Gluteus medius; PCA, Principal component analysis; MEP, Motor evoked potential; TMS, Transcranial magnetic stimulation; SIS, Stroke impact scale; TUG, Timed-up-and-go.

## Competing interests

The authors declare that they have no competing interests.

## Authors’ contributions

CK and RR contributed equally. All authors contributed to the conception of the study. CK, RR, and SSK collected, analyzed, and interpreted the data. CK performed the training. All authors contributed to the writing of the manuscript. All authors read and approved the final version of the manuscript.
